# A meta-analysis of highly active anti-retroviral therapy for treatment of plasmablastic lymphoma

**DOI:** 10.4103/0256-4947.60517

**Published:** 2010

**Authors:** Bing Guan, Xinhau Zhang, Henhui Ma, Hangbo Zhou, Xiaojun Zhou

**Affiliations:** From the Clinical School of Medical College of Nanjing University and Nanjing Jinling Hospital, Department of Pathology, Nanjing 210002, China

## Abstract

**BACKGROUND AND OBJECTIVES:**

Plasmablastic lymphoma is a recently described B-cell derived lymphoma. The prognosis of plasmablastic lymphoma patients is usually poor. We performed a systematic review of the literature on the use of highly active anti-retroviral therapy (HAART) and the prognosis of plasmablastic lymphoma.

**METHODS:**

A comprehensive search of relevant databases, including Medline, Embase, the Cochrane Controlled Trials Register, the Cochrane Library, and the Science Citation Index yielded ten randomized controlled trials. Trials were divided into two groups according to therapy. The rates of plasmablastic lymphoma were analyzed using a fixed-effects model. Sensitivity analyses (on publication type, statistical model) were performed to further detect and evaluate clinically significant heterogeneity. Tests of survival for plasmablastic lymphoma were also performed by using Kaplan-Meier method.

**RESULTS:**

Meta-analysis result showed that the prognosis of plasmablastic lymphoma patients was statistically different in the patients receiving HAART in addition to chemotherapy and/or radiotherapy than in the patients receiving the chemotherapy and/or radiotherapy alone (pooled relative risk=3.04; *P*=.03). Survival analyses also displayed a statistically significant difference (χ^2^=6.22, *P*=.013).

**CONCLUSION:**

HAART in addition to chemotherapy and/or radiotherapy is effective in improving the prognosis of plasmablastic lymphoma. However, the small sample sizes increase the likelihood of bias in the studies in this meta-analysis, and therefore, the results should be taken cautiously.

Plasmablastic lymphoma (PBL) is a rare variant of a diffuse, large B-cell lymphoma, which typically presents in the oral cavity in immunodeficient patients.^1^ More recently, the World Health Organization (WHO) has classified PBL, accepted as a new entity in the WHO classification of tumors of the hematolpoietic lymphoid tissues (4th edition), as an AIDS-related illness. According to the definition of WHO, PBL is a diffuse proliferation of large neoplastic cells, most of which similar to B immunoblasts, but in which all tumor cells have the immunophenotype of plasma cells (CD138 and VS38c positive).^2^–^4^ PBL accounts for approximately 2.6% of all HIV-related non-Hodgkin lymphomas (NHL).^5^ About 76% of PBLs are reported to be infected by EBV and 17% to 37% by Kaposisarcoma-associated herpesvirus (HHV8).^1^,^6^–^8^

The prognosis of PBL patients is usually poor, regardless of the site of origin, and the factors influencing prognosis are not clear. The clinical course is very aggressive with most of patients dying in the first year after diagnosis.^7^,^9^ After a review of the literature, we concluded that highly active anti-retrovirus therapy (HAART) might be an important influencing factor on the prognosis of PBL. Therefore, we performed a meta-analysis to assess the effects of HAART on the prognosis of PBL.

## METHODS

Only randomized controlled trials (RCTs) on the prognosis of PBL that included patients receiving treatment with HAART were eligible for inclusion in this study. All patients had to be diagnosed as having PBL confirmed by pathology, including some variants of PBL who were HIV negative. Patients with other variants of diffuse large B-cell lymphoma with plasmablastic features were not included in this study. Inclusion criteria for each study in the main analysis were: (1) a minimal clinical follow-up of 24 hours; (2) a minimal sample size of 2 per trial; (3) patients receiving chemotherapy and/or radiotherapy and/or HAART; (4) comparison patients not receiving HAART or patients receiving chemotherapy and/or radiotherapy alone.

We used the following sources for the identification of trials: the Cochrane Central Register of Controlled Trials, the Cochrane Library, PubMed, Embase, Science Citation Index and meeting abstracts, up to December 2008. Key words were ‘plasmablastic lymphoma’, ‘plasmablastic lymphomas’, ‘highly active anti-retrovirous therapy’, ‘HAART’. The search terms were MeSH terms and the search was limited to RCTs in English. Two reviewers (Guan and Zhou) independently checked the titles, abstract sections and keywords of every record retrieved. Full articles were retrieved for further assessment when the information suggested the studies were eligible. References in papers identified in the electronic search were manually searched for relevant trials not included in electronic databases. Each was imported into a bibliographic database EndNote (Version X1) and merged into one core database. The contents of that database were exported to the computer program Review Manager (Version 5.0 for Windows; the Cochrane Collaboration, Oxford, UK, *http://www.cc-ims.net/revman*).

Two reviewers extracted data on intervention and outcomes independently, using a pre-tested data extraction form that was adapted from a standard form provided by the review group. The data extraction form included general information, study characteristics, information on participants, interventions, baseline characteristics and measurements and outcomes. Differences in data extraction were resolved by consensus, referring back to the original article. If necessary, information was sought from the authors of the original studies. The included studies were divided into two groups according to whether they received chemotherapy or radiotherapy or HAART vs. non-therapy (subgroup 1), and to whether they received HAART in addition to chemotherapy and/or radiotherapy vs. receiving chemotherapy and/or radiotherapy alone (subgroup 2).

Assessment of each trail was performed by two investigators (Guan and Zhou) independently. Disagreement was resolved by discussion between the two reviewers with consensus or with consultation of a third reviewer (Zhang). In particular, the following quality criteria were assessed: randomization procedure, allocation concealment, method of blinding, handling of drop-outs (intention-to-treat analysis), quantity of dropouts, selective (skewed) dropout, and method of blinding outcome assessment (if applicable). Quality of the trials were assessed by the Jadad quality scale,^12^,^13^ a composite scoring system that includes randomization, concealment, and patient withdrawal and dropout. Trials with a score ≥3 were considered of high quality.

Dichotomous data were expressed as odds ratios (OR) or risk ratio (RR). Heterogeneity among the studies was examined using the DLQ statistic.^10^ Pooled OR, RR and 95% confidence interval (CI) was calculated using the Mantel-Haenszel fixed-effects model when no statistically significant heterogeneity was detected. A random effects model was employed using the DerSimonian and Laird (DL) method if there was statistically significant heterogeneity. The chi-square statistic was set at α=0.1, with *P*<.1 indicating a statistically significant difference. The Z score was set at α=0.05, with *P*<.05 indicating a statistically significant difference.

Publication bias was estimated by examining the relationship between the treatment effects and the standard error of the estimate (S.E log RR) using a funnel plots. Quantification of the effect of heterogeneity would be assessed by means of *I* squared, ranging from 0% to 100% including its 95% confidence interval (CI) with significance set at *P*>50%.^11^ *I*-squared demonstrates the percentage of total variation across studies due to heterogeneity and was used to judge the consistency of evidence. The analyses were done with the Review Manager (Version 5.0 for Windows; the Cochrane Collaboration, Oxford, UK, *http://www.cc-ims.net/revman*). The statistically significant differences in each control group between subgroups were analyzed using the chi-square test, with *P*<.05 indicating statistically differences. Test of survival for HAART on the prognosis of PBL were also performed by using Kaplan-Meier method, with *P*<.05 indicating statistically significant differences. The date were processed by use of SPSS 16.0 (SPSS, Chicago, IL).

Several sensitivity analyses were performed to investigate the possible influence of the predefined quality criteria, including the statistical model (random-effects vs. fixed-effects). OR values were calculated for the sensitivity analyses. Several methods were employed to assess sensitivity. The effect of publication type was examined by excluding trials published in the form of abstracts only. The effect of study quality was examined by excluding trials with a Jadad score <3. The effect of sample size was examined by excluding trials with sample size <2.

## RESULTS

**Figure 1:**
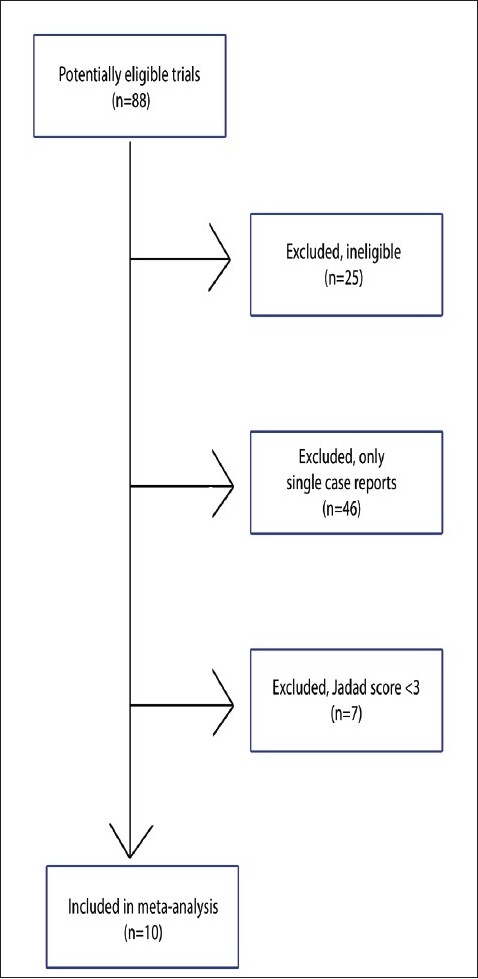
Study flow diagram of included and excluded studies.

**Figure 2:**
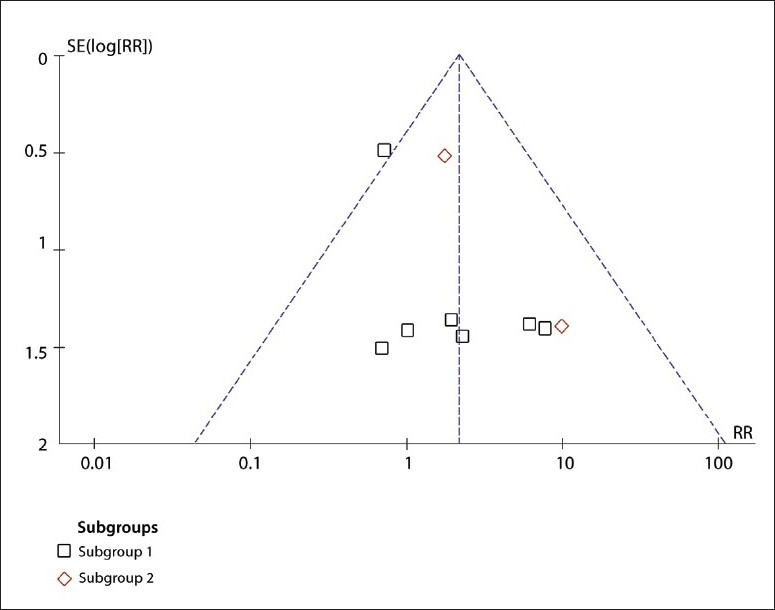
Funnel plot for the detection of publication bias for subgroups indicating no publication bias (broken lines are 95% confidence limits).

A total of 88 studies were retrieved (Figure 1). Of these, 25 were irrelevant and excluded and 46 were excluded because they were only case reports. Seven studies were excluded because of poor data (Jadad score <3). Ten studies were included in our analysis (Table 1).^1^,^7^,^14^–^19^ Subgroup 1 included eight trials. Subgroup 2 included two trials. A funnel plots suggested there was no publication bias (Figure 2).

**Table 1:** Characteristics of the 10 studies in the meta-analysis.

Study	Sample size	Research design	Jadad score
Subgroup 1			
Chetty 2003^14^	4	Unclear	3
Cioc 2004^15^	4	Unclear	3
Delecluse 1997^1^	16	RCT	4
Dong 2005^7^	14	RCT	3
Folk 2006^16^	5	RCT	3
Gaidano 2002^17^	12	RCT	4
Reidal 2008^18^	5	RCT	3
Teruya-Feldstein 2005^19^	12	RCT	4
Subgroup 2			
Gaidano 2002^17^	10	RCTs	4
Teruya-Feldstein 2005^19^	11	RCTs	4

Subgroup 1: Chemotherapy or radiotherapy or HAART vs. no therapy

Subgroup 2: HAART in addition to chemotherapy and/or radiotherapy vs. chemotherapy and/or radiotherapy alone.

Methodological quality: All studies were double-blinded; drop-out rates and randomization methods were clearly reported, but allocation concealment was unclear in every report.

### Primary outcomes

**Figure 3:**
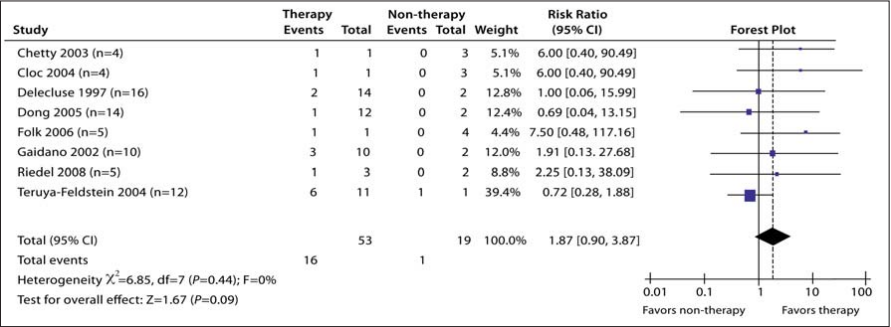
Forest plot for results in subgroup 1 studies, indicating no statistically significant differences between chemotherapy or radiotherapy or HAART vs non-therapy (size of data points represents sample size of study).

**Figure 4:**
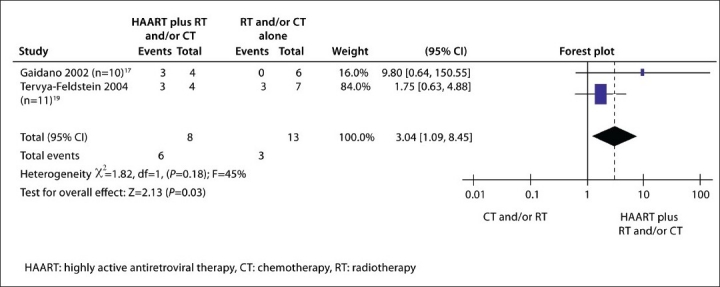
Forest plots for results of meta-analysis in subgroup 2 studies, indicating statistically significant differences for HAART in addition to chemotherapy and/or radiotherapy vs chemotherapy and/or radiotherapy alone (size of data points represents sample size of study).

Ten studies on PBL involving 93 patients were identified. No statistically significant heterogeneity existed in either subgroup 1 (*P*=.44) (Figure 3) or in subgroup 2 (*P*=.18) (Figure 4). In subgroup 1, the results showed no statistically significant differences in the patients receiving chemotherapy or radiotherapy or HAART than in the patients receiving non-therapy (30.2%, 16/53 vs. 5.3%, 1/19; pooled RR=1.87; 95% CI: 0.90-3.87; *P*=.09) on the prognosis of PBL. In subgroup 2, the results indicated statistically significant differences in patients receiving HAART in addition to chemotherapy and/or radiotherapy compared with patients receiving chemotherapy and/or radiotherapy alone (75%, 6/8 vs. 23.1%, 3/13; pooled RR=3.04; 95% CI: 1.09-8.45; *P*=.03) on the prognosis of PBL. No statistically significant differences were detected in the prognosis of PBL between the controls in either subgroup (5.26%, 1/19 vs. 23.08, 3/13%, χ^2^=1.70, *P*=.19).

### Sensitivity analyses

We selected a random-effects model for the analysis. Overall, the estimates of effect on prognosis of PBL were virtually identical for the sensitivity analyses and the meta-analysis (Table 2).

**Table 2:** Sensitivity analysis for the possible influence of the predefined quality criteria.

Method	Subgroup	Patients	OR (95%CI)	Z	*P*	Heterogeneity		
						c^2^	*P*	I^2^ (%)
Full text of publication	1	72	2.52 (0.78, 8.16)	1.54	.12	5	.73	0
	2	21	8.91 (1.16, 68.54)	2.1	.04	0.82	.37	0
Random-effect model	1	72	2.52 (0.68, 9.30)	1.38	.17	5	.66	0
	2	21	8.67 (1.03, 73.20)	1.98	.05	0.82	.37	0

**Total events**		**93**	**3.49 (1.27, 9.59)**	**2.43**	**.02**	**6.76**	**.66**	**0**

### Survival analyses

**Figure 5:**
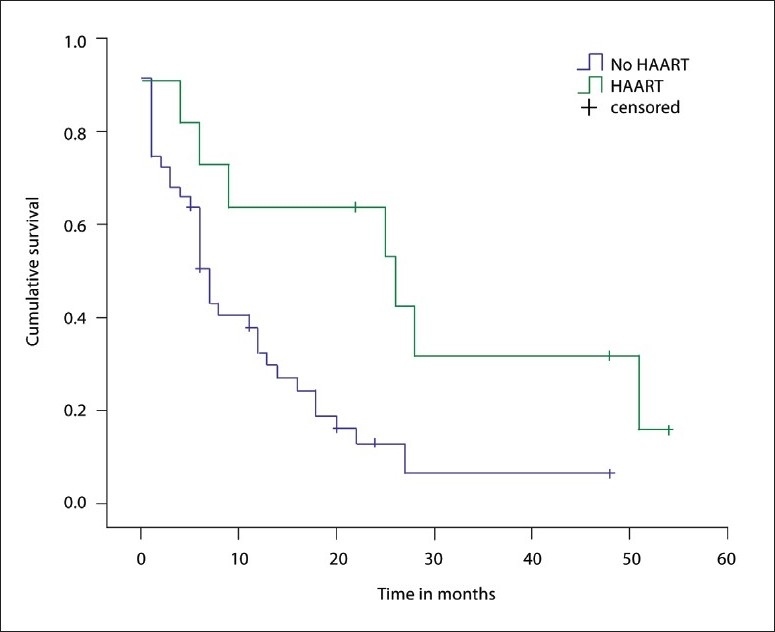
Kaplan-Meier survival analysis plots showing statistically significant differences between intervention groups and controls (χ^2^=6.22, degree of freedom=1, *P*=.013, log rank test with two-sided *P* values).

Survival analyses were performed for the comparison of HAART in addition to chemotherapy and/or radiotherapy vs. the chemotherapy and/or radiotherapy alone, and for the comparison of HIV-infected vs. non-HIV infected patients. In the comparison of HAART, statistically significant differences on the prognosis of PBL were detected between the patients receiving HAART in addition to chemotherapy and/or radiotherapy and the patients receiving the chemotherapy and/or radiotherapy only (χ^2^=6.22, degree of freedom=1, *P*=.013, log rank test with two-sided P values) (Figure 5). In the comparison of HIV vs non-HIV patients, no statistically significant differences was detected in the prognosis of PBL (χ^2^=2.49, degree of freedom=1, *P*=.114, log rank test with two-sided *P* values).

## DISCUSSION

The factors influencing the prognosis of PBL are not clear. Castillo et al^20^ studied 112 published cases and found that EBV status, primary site of involvement, and clinical stage failed to show an association with survival using log-rank tests with two-sided P values. However, he did not perform a meta-analysis. On the other hand, there is no unified and effective agreement on the therapy for PBL.^18^ Therapy depends on the clinical stage and the clinical symptoms caused by HIV infection. Moreover, there is no information on how choice of therapy relates to prognosis of PBL.^18^,^20^–^23^ To clarify this question, we used the Cochrane systematic review method.

Our meta-analysis showed that HAART combined with chemotherapy and/or radiotherapy was more effective in improving the prognosis of PBL patients than chemotherapy and/or radiotherapy alone. However, The 95% CIs in some studies were very wide and the sample sizes small, which indicates a lack of precision in the estimate of relative risk. The small sizes of the trials might introduce bias in the results of the meta-analysis. There might also be a language bias, since only trials reported in English were included. The significant results in the survival analyses support the hypothesis that the addition of HAART to therapy has a favorable effect on prognosis, but the limitations associated with retrospectively accumulated data make it hard to draw firm conclusions. Therefore, the results should be interpreted cautiously. Larger, prospective studies are needed to further elucidate survival advantages for treatment that includes HAART.

## References

[1] Delecluse HJ, Anagnostopoulos I, Dallenbach F, Hummel M, Marafioti T, Schneider U, Huhn D (1997). Plasmablastic lymphomas of the oral cavity: a new entity associated with the human immunodeficiency virus infection. Blood.

[2] Kim JE, Kim YA, Kim WY, Kim CW, Ko YH, Lee GK, Choi SJ, Jeon YK (2009). Human immunodeficiency virus-negative plasmablastic lymphoma in Korea. Leuk Lymphoma.

[3] Kane S, Khurana A, Parulkar G, Shet T, Prabhash K, Nair R (2009). Minimum diagnostic criteria for plasmablastic lymphoma of oral/sinonasal region encountered in a tertiary cancer hospital of a developing country. J Oral Pathol Med.

[4] Bose P, Thompson C, Gandhi D, Ghabach B, Ozer H (2009). AIDS-related plasmablastic lymphoma with dramatic, early response to bortezomib. Eur J Haematol.

[5] Carbone A (2002). AIDS-related non-Hodgkin's lymphomas: from pathology and molecular pathogenesis to treatment. Hum Pathol.

[6] Rafaniello Raviele P, Pruneri G, Maiorano E (2009). Plasmablastic lymphoma: a review. Oral Dis.

[7] Dong HY, Scadden DT, de Leval L, Tang Z, Isaacson PG, Harris NL (2005). Plasmablastic lymphoma in HIV-positive patients: an aggressive Epstein-Barr virus-associated extramedullary plasmacytic neoplasm. Am J Surg Pathol.

[8] Carbone A, Gloghini A, Larocca LM, Capello D, Pierconti F, Canzonieri V (2001). Expression profile of MUM1/IRF4, BCL-6, and CD138/syndecan-1 defines novel histogenetic subsets of human immunodeficiency virus-related lymphomas. Blood.

[9] Carbone A, Gloghini A, Gaidano G (2004). Is plasmablastic lymphoma of the oral cavity an HHV-8-associated disease?. Am J Surg Pathol.

[10] DerSimonian R, Laird N (1986). Meta-analysis in clinical trials. Control Clin Trials.

[11] Higgins JP, Thompson SG (2002). Quantifying heterogeneity in a meta-analysis. Stat Med.

[12] Kjaergard LL, Villumsen J, Gluud C (2001). Reported methodologic quality and discrepancies between large and small randomized trials in meta-analyses. Ann Intern Med.

[13] Jadad AR, Moore RA, Carroll D, Jenkinson C, Reynolds DJ, Gavaghan DJ (1996). Assessing the quality of reports of randomized clinical trials: is blinding necessary?. Control Clin Trials.

[14] Chetty R, Hlatswayo N, Muc R, Sabaratnam R, Gatter K (2003). Plasmablastic lymphoma in HIV+ patients: an expanding spectrum. Histopathology.

[15] Cioc AM, Allen C, Kalmar JR, Suster S, Baiocchi R, Nuovo GJ (2004). Oral plasmablastic lymphomas in AIDS patients are associated with human herpesvirus 8. Am J Surg Pathol.

[16] Folk GS, Abbondanzo SL, Childers EL, Foss RD (2006). Plasmablastic lymphoma: a clinicopathologic correlation. Ann Diagn Pathol.

[17] Gaidano G, Cerri M, Capello D, Berra E, Deambrogi C, Rossi D (2002). Molecular histogenesis of plasmablastic lymphoma of the oral cavity. Br J Haematol.

[18] Riedel DJ, Gonzalez-Cuyar LF, Zhao XF, Redfield RR, Gilliam BL (2008). Plasmablastic lymphoma of the oral cavity: a rapidly progressive lymphoma associated with HIV infection. Lancet Infect Dis.

[19] Teruya-Feldstein J (2005). Diffuse large B-cell lymphomas with plasmablastic differentiation. Curr Oncol Rep.

[20] Castillo J, Pantanowitz L, Dezube BJ (2008). HIV-associated plasmablastic lymphoma: lessons learned from 112 published cases. Am J Hematol.

[21] Mani D, Guinee DG, Aboulafia DM (2008). AIDS-associated plasmablastic lymphoma presenting as a poorly differentiated esophageal tumor: a diagnostic dilemma. World J Gastroenterol.

[22] Goedhals J, Beukes CA, Hardie D (2008). HHV8 in plasmablastic lymphoma. Am J Surg Pathol.

[23] Carbone A, Gloghini A (2008). Plasmablastic lymphoma: one or more entities?. Am J Hematol.

